# FebriDx host response point-of-care testing improves patient triage for coronavirus disease 2019 (COVID-19) in the emergency department

**DOI:** 10.1017/ice.2021.531

**Published:** 2022-08

**Authors:** Christopher T. Mansbridge, Alex R. Tanner, Kate R. Beard, Florina Borca, Hang T.T. Phan, Nathan J. Brendish, Stephen Poole, Christopher Hill, Michael Kiuber, Robert Crouch, Daniel Waddington, Tristan W. Clark

**Affiliations:** 1 Department of Infection, University Hospital Southampton NHS Foundation Trust, Southampton, United Kingdom; 2 National Institute for Health Research (NIHR) Southampton Biomedical Research Centre, University Hospital Southampton NHS Foundation Trust, Southampton, United Kingdom; 3 Clinical Informatics Research Unit, University of Southampton, Southampton, United Kingdom; 4 School of Clinical and Experimental Sciences, Faculty of Medicine, University of Southampton, Southampton, United Kingdom; 5 NIHR Post Doctoral Fellowship Programme, University Hospital Southampton NHS Foundation Trust, Hampshire, United Kingdom; 6 Department of Emergency Medicine, University Hospital Southampton NHS Foundation Trust, Southampton, United Kingdom; 7 Health Sciences, Faculty of Environmental and Life Sciences, University of Southampton, Southampton, United Kingdom

## Abstract

**Objectives:**

Patients presenting to hospital with suspected coronavirus disease 2019 (COVID-19), based on clinical symptoms, are routinely placed in a cohort together until polymerase chain reaction (PCR) test results are available. This procedure leads to delays in transfers to definitive areas and high nosocomial transmission rates. FebriDx is a finger-prick point-of-care test (PoCT) that detects an antiviral host response and has a high negative predictive value for COVID-19. We sought to determine the clinical impact of using FebriDx for COVID-19 triage in the emergency department (ED).

**Design:**

We undertook a retrospective observational study evaluating the real-world clinical impact of FebriDx as part of an ED COVID-19 triage algorithm.

**Setting:**

Emergency department of a university teaching hospital.

**Patients:**

Patients presenting with symptoms suggestive of COVID-19, placed in a cohort in a ‘high-risk’ area, were tested using FebriDx. Patients without a detectable antiviral host response were then moved to a lower-risk area.

**Results:**

Between September 22, 2020, and January 7, 2021, 1,321 patients were tested using FebriDx, and 1,104 (84%) did not have a detectable antiviral host response. Among 1,104 patients, 865 (78%) were moved to a lower-risk area within the ED. The median times spent in a high-risk area were 52 minutes (interquartile range [IQR], 34–92) for FebriDx-negative patients and 203 minutes (IQR, 142–255) for FebriDx-positive patients (difference of −134 minutes; 95% CI, −144 to −122; *P* < .0001). The negative predictive value of FebriDx for the identification of COVID-19 was 96% (661 of 690; 95% CI, 94%–97%).

**Conclusions:**

FebriDx improved the triage of patients with suspected COVID-19 and reduced the time that severe acute respiratory coronavirus virus 2 (SARS-CoV-2) PCR-negative patients spent in a high-risk area alongside SARS-CoV-2–positive patients.

The management of the coronavirus disease 2019 (COVID-19) pandemic is hindered by long delays in diagnosis. Due to the limited availability of single-room accommodation in UK hospitals, patients are routinely placed in a cohort together, based on clinical symptoms, until diagnostic test results are available.^1^ This procedure results in delays in transfers to definitive clinical areas and high rates of nosocomial transmission.^2,3^ Although molecular point-of-care tests (PoCTs) can dramatically reduce time to diagnosis,^4^ the availability of such tests has remained limited. Low availability and high cost have prevented their widespread routine use thus far.

Emergency departments (EDs) are busy and often overcrowded places that represent a high-risk clinical area for transmission of severe acute respiratory coronavirus virus 2 (SARS-CoV-2) among patients. The unsuitability of current UK EDs for managing patients in the context of a pandemic with a highly transmissible infectious agent has been recognized at the national level.^5^ In addition to the limited physical space in EDs, the lack of real-time diagnostic results compounds the problem and leads to poor patient flow; patients deemed at high risk of having COVID-19 based on symptoms are usually nursed together in ‘high-risk’ cohort areas until they are admitted or discharged.^1^ The lack of single-occupancy rooms or adequately distanced bay areas in most EDs means that patients without COVID-19 in these high-risk cohort areas are at great risk of acquiring the infection from neighboring SARS-CoV-2–positive patients before results are available.

FebriDx (Lumos Diagnostics, Sarasota, FL) is a Conformitè Europëenne (CE)-marked lateral flow immunoassay PoCT originally designed to differentiate between bacterial and viral respiratory infections by detecting two host-response proteins, myxovirus resistance protein A (MxA) and C-reactive protein (CRP), in finger-prick blood samples.^6–10^ MxA is a specific marker of interferon-induced antiviral host response. Studies performed during the first wave of the pandemic demonstrated that MxA detection has high sensitivity and negative predictive value for identifying patients with COVID-19.^11–13^ FebriDx is a low-cost, analyzer-free, easy-to-use PoCT device that returns results in 10 minutes. As highlighted in a recent NICE Medtech briefing,^14^ FebriDx could be used to improve risk stratification of patients with suspected of COVID-19 in EDs. However, no studies have evaluated its clinical impact in this setting.

We sought to address this high-priority evidence gap by conducting an observational study evaluating the clinical impact of using FebriDx to improve the triage of patients with possible COVID-19 in our ED.

## Methods

### Setting

This single-center retrospective observational study was conducted in the ED of the University Hospital Southampton NHS Foundation Trust (UK), a large, acute-care teaching hospital serving a secondary care population of ∼650,000 people. In this study, we used routinely collected, anonymized data. Study approval was granted by the University Hospital Southampton NHS Foundation Trust and the trust data protection office. Local research and development governance officers confirmed that research ethics committee review was not required.

### Intervention

FebriDx is a self-contained lateral-flow–based PoCT that detects two host-response proteins, CRP and MxA. The manufacturer’s instructions for use are available at www.febridx.com/how-to-use#testing. Briefly, the patient’s skin is punctured by an integral lancet and 5 µL blood is drawn into the collection tube by placing it against a blood drop. Blood is then transferred to the lateral flow section of the device and reagents are released by pressing a button. After 10 minutes, visual inspection reveals the presence or absence of three lines: a grey CRP line (top; detection threshold 20 mg/L), a red MxA line (middle; detection threshold 40 ng/mL), and a blue control line (bottom).^6^

### Implementation

**Fig. 1.**
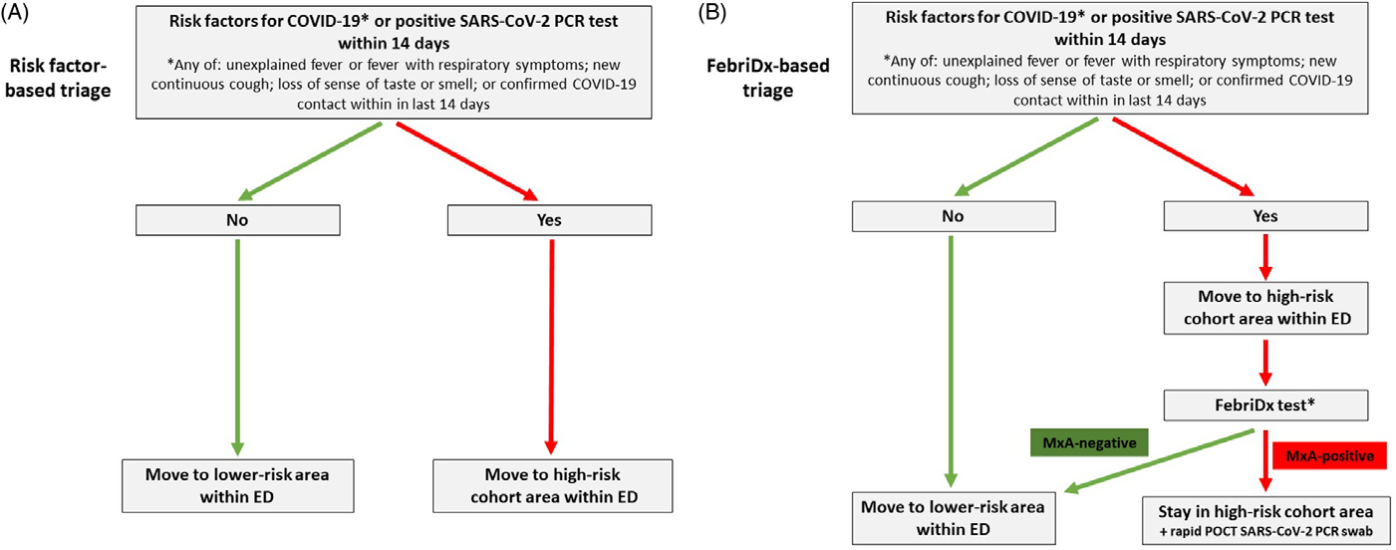
Emergency department COVID-19 risk triage algorithm (A) prior to study based on risk factors only and (B) during study based on risk factors and FebriDx result. *FebriDx testing was not undertaken in patients with immunosuppression, symptoms for >14 days, or with a positive SARS-CoV-2 PCR test within 14 days, and asymptomatic COVID-19 contacts. These patients stayed in the high-risk cohort area until discharged or admitted.

Prior to this study, patients presenting to the ED were placed in a cohort in the high-risk area solely based on (1) the presence of a positive SARS-CoV-2 PCR test within the previous 14 days or (2) risk factors for COVID-19 including unexplained fever or fever with respiratory symptoms; new continuous cough; loss of sense of taste or smell; or known contact with a patient with confirmed COVID-19.^15^ Patients who met any of these criteria were immediately moved to the high-risk cohort area and patients who did not were managed in lower-risk areas within the ED. The high-risk cohort area was a departmental area with limited bed space but with additional infection control precautions to prevent cross infection, including floor-to-ceiling solid plastic screens between patient beds. Personal protective equipment in accordance with Public Health England guidance was worn in all areas.^16^ Patients remained in these cohort areas until either they were discharged home or they were admitted to speciality areas within the hospital (Fig. 1A).

Following a period of training and pilot testing, a FebriDx-based COVID-19 risk triage system was implemented in the ED on September 22, 2020. Once patients with risk factors for COVID-19 arrived in the high-risk cohort area, trained personnel applied the FebriDx test after obtaining verbal consent. Patients aged <18 years, with immunosuppression, symptom duration >14 days, and patients without COVID-19 symptoms were not tested using FebriDx because the diagnostic accuracy of FebriDx for identifying COVID-19 in these patient groups has not been established. Patients with confirmed COVID-19, diagnosed by PCR testing in the preceding 14 days, also were not tested using FebriDx, according to local protocols. After FebriDx results were available, patients who did not have a detectable antiviral host response (ie, MxA negative) were recategorized as low risk for COVID-19 and were moved to a lower-risk area within the ED (Fig. 1B).

Patients with a detected antiviral host response (ie, MxA positive) remained in the high-risk cohort area, and those who required hospital admission were then tested for SARS-CoV-2 and other respiratory viruses using rapid multiplex PCR testing. Patients who were SARS-CoV-2 PCR positive were transferred directly to COVID-19–positive wards, bypassing speciality assessment areas and reducing the risk of further exposure to other patients. Patients who required hospital admission with a negative MxA who were moved from the high-risk cohort area to lower-risk areas in the ED were subsequently tested for SARS-CoV-2 using laboratory PCR when they arrived in the relevant speciality admissions area. In accordance with hospital policy, patients who were discharged from the ED were not routinely tested for SARS-CoV-2 by PCR but were given advice about risk and isolation.

### Clinical data

We reviewed routinely collected data on all patients managed in the high-risk cohort area and those managed in the ‘majors’ area (ie, a lower-risk area for patients with major illness) in the ED during the study period. We collected the following data: demographic data and comorbidities, times of arrival and transfer to different departmental areas, time patients left the department and their discharge destination, FebriDx results (if tested), and PCR results for SARS-CoV-2 (if tested).

### Outcome measures

The primary outcome of interest was the time FebriDx MxA-negative patients spent in the high-risk cohort area compared to MxA-positive patients. Secondary outcomes of interest included proportion of patients moved to low risk areas, the time patients spent in low-risk areas within ED, total time spent in the ED, time to PCR result according to FebriDx result, the number and proportion of patients who were correctly and incorrectly moved to lower-risk ED areas based on subsequent SARS-CoV-2 PCR results, and the diagnostic accuracy (sensitivity, specificity, negative and positive predictive values, and overall diagnostic accuracy) of FebriDx for COVID-19 compared to the reference standard of PCR.

### Statistical analysis

Analysis was based on patient ‘episodes’ rather than individual patients. If the same patient attended the ED more than once during the study period, they were included in analyses again and were counted as separate patient episodes. Analyses were carried out using Prism version 7.0 software (GraphPad, La Jolla, CA). Baseline characteristics were summarized for patients tested using FebriDx for whom data were available as were FebriDx MxA results. The primary outcome measure was compared between FebriDx MxA-positive and MxA-negative patients using the Mann-Whitney *U* test. For the secondary outcomes, the Mann-Whitney *U* test was used to compare continuous data (eg, time to PCR result). Differences in median times and their 95% confidence intervals (CIs) were calculated using the Hodges–Lehmann estimate. Differences in proportions were assessed using the χ^2^ test or the Fisher exact test, as appropriate depending on group size. For measures of diagnostic accuracy, sensitivity, specificity, predictive values, and likelihood ratios were calculated for FebriDx MxA detection for the identification of COVID-19, compared with the reference standard of SARS-CoV-2 PCR. Time-to-event analysis data were analyzed using the *lifelines* package in Python 3.7 software and were compared using the log-rank test. The 95% CIs were calculated using Prism defaults.

## Results

**Fig. 2.**
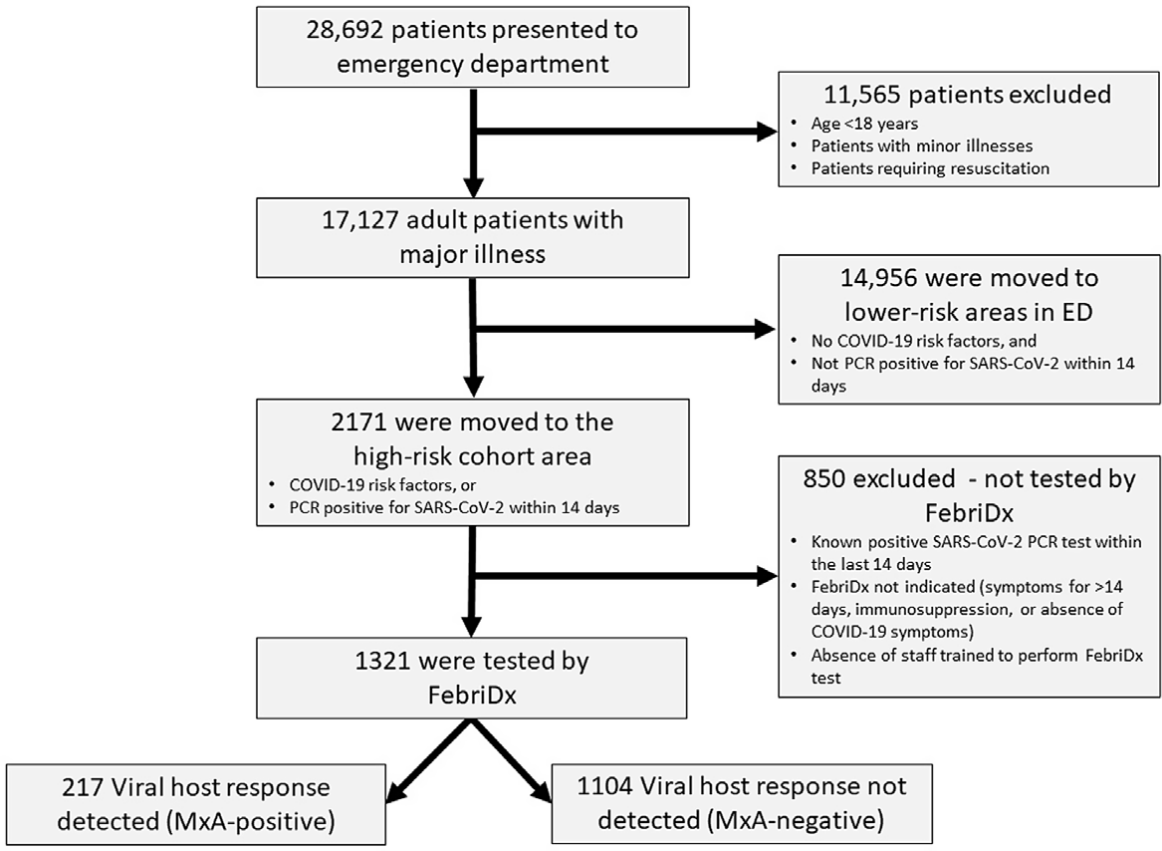
Flow of participants.

Between September 22, 2020, and January 7, 2021, 28,692 patients presented to the ED, and 17,127 patients presented with major illness (ie, were triaged to the ‘majors’ section of the ED at arrival). Overall, 2,171 (13%) fulfilled criteria for possible COVID-19 or were PCR positive for SARS-CoV-2 within the previous 14 days and were therefore moved to the high-risk cohort area. Furthermore, 14,956 (87%) did not meet these criteria, and they were moved to a lower-risk area within the department. The flow of study participants in the study is shown in Figure 2.

### Patient and departmental flow using FebriDx-based triage

**Fig. 3.**
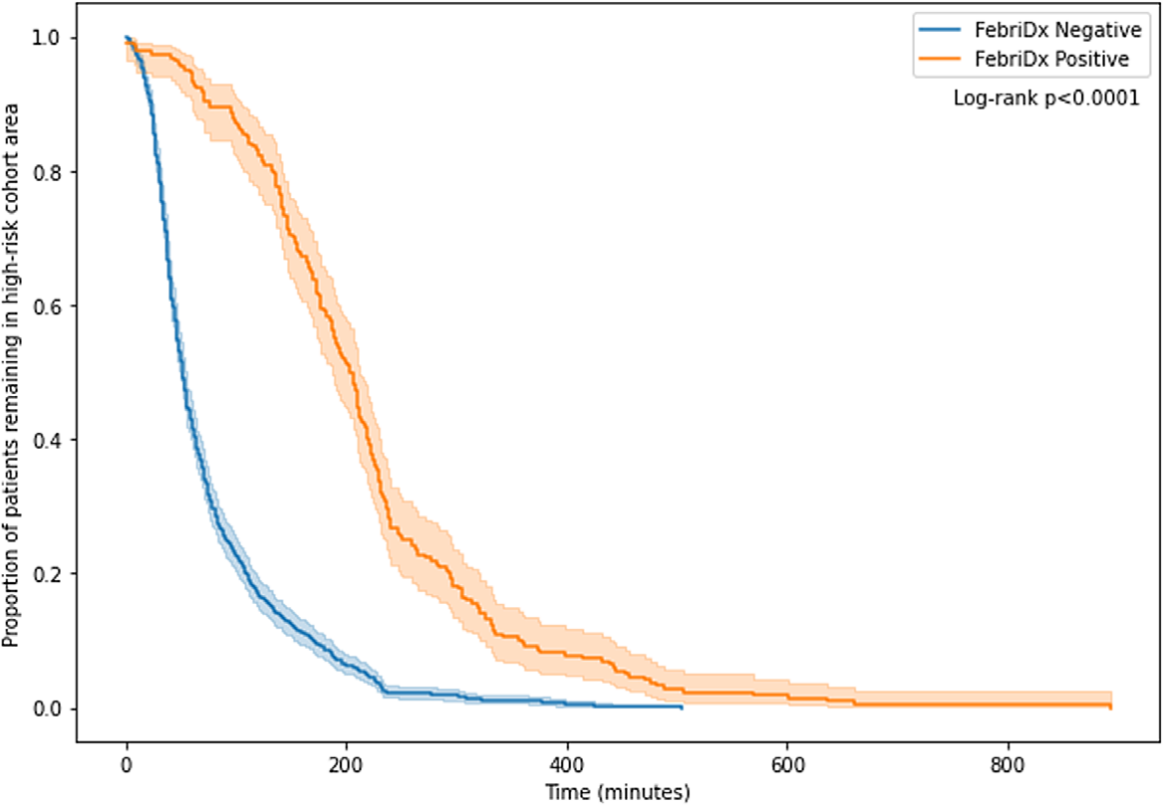
Time-to-event curve for time to leaving high-risk cohort area, by FebriDx result.

Overall, 1,321 (61%) of the 2,171 patients triaged to the high-risk pathway were tested using FebriDx. 850 (39%) were not tested for the following reasons: (1) PCR positive for SARS-CoV-2 with 14 days; (2) FebriDx not indicated (due to immunosuppression, symptoms for >14 days, or the absence of COVID-19 symptoms); or (3) unavailability of staff trained to perform FebriDx testing. The median time from presentation to ED and FebriDx testing was 30 minutes (interquartile range [IQR], 19–45). Also, 17 FebriDx tests (1.3%) had to be repeated due to absence of control line or operator error. Of those tested using FebriDx, 217 (16%) of 1,321 patients were MxA positive and 1,104 (84%) were MxA negative. Compared to MxA-negative patients, higher proportions of FebriDx MxA-positive patients were male, were of Asian ethnicity, and had cardiovascular disease or malignancy. Baseline demographic and clinical characteristics for all patients tested by FebriDx and according to result are shown in Table 1. Of 1,104 MxA-negative patients, 865 (78%) were moved from a high-risk cohort area to a lower-risk area within the ED (the remainder stayed in the high-risk area). Of 217 MxA-positive patients, 210 (97%) remained within the high-risk area for the duration of their stay in ED. A review of patient records did not clarify why some patients were moved against protocol; however, it is likely due to departmental flow and bed occupancy pressures. The median time spent in high-risk areas was 52 minutes (IQR, 34–92) for FebriDx MxA-negative patients and 203 minutes (IQR, 142–255) for FebriDx MxA-positive patients (difference of −134 minutes; 95% CI, −144 to −122; *P* < .0001). The time-to-event analysis for time to leaving the high-risk cohort area according to FebriDx result is shown in Figure 3. The details of patients moved from the high-risk cohort area to a lower-risk area and the time spent in each area are shown in Table 2.

**Table 1.** Baseline Demographics and Clinical Characteristics for All FebriDx–Tested Patients and by FebriDx MxA Result

Variable	All Patients (n = 1,321), No. (%)	MxA-Positive Patients (n = 217), No. (%)	MxA-Negative Patients (n = 1104), No. (%)	Between-Group Difference (95% CI)^a^	*P*-Value^a^
Age, median y (IQR)	62 (40–78)	63 (42–75)	62 (38–79)	−1 (−4 to 3)	.865
**Sex**	1,321	217	1,104		
Male	672 (51)	127 (59)	545 (49)	−9 (−16 to −2)	.0143
Female	649 (49)	90 (41)	559 (51)	…	…
**Ethnicity**	1,220	194	1,026		
White British	1,042 (85)	155 (80)	887 (86)	7 (0 to 12)	.026
White other	62 (5)	10 (5)	52 (5)	0 (−3 to 5)	1.0
Black	10 (1)	2 (1)	8 (1)	0 (−1 to 3)	.665
Asian	75 (6)	22 (11)	53 (5)	−6 (−12 to −2)	.0028
Other	31 (3)	5 (3)	26 (3)	0 (−2 to 4)	1.0
**Comorbidities**	976	163	813		
Hypertension	347 (36)	65 (40)	282 (35)	−5 (−14 to 3)	.211
Diabetes	195 (20)	39 (24)	156 (19)	−5 (−13 to 2)	.165
Cardiovascular disease	346 (35)	77 (47)	269 (33)	−14 (−23 to −6)	.0009
Chronic respiratory disease	338 (35)	49 (30)	289 (36)	5 (−2 to 14)	.207
Chronic kidney disease	133 (14)	27 (17)	106 (13)	−4 (−11 to 2)	.230
Chronic liver disease	42 (4)	8 (5)	34 (4)	−1 (−6 to 2)	.673
Malignancy	124 (13)	29 (18)	95 (12)	−6 (−13 to 0)	.039
Dementia	69 (7)	8 (5)	61 (8)	3 (−7 to 8)	.314

Note. MxA, myxovirus resistance protein. CI, confidence interval; IQR, interquartile range.

a Between FebriDx MxA-positive and -negative groups.

**Table 2.** Details of Patient Moves Within the Emergency Department and Time to PCR Results for FebriDx MxA-Positive and MxA-Negative Patients

Variable	MxA-Positive Patients (n = 217))	MxA-Negative Patients (n = 1,104)	Difference (95% CI)^a^	*P* Value
Patients moved from high-risk cohort area to lower-risk areas of ED, no. (%)	7 (3)	865 (78)	75% (72%–80%)	<.0001
Total time in ED, min (IQR)	237 (200–329)	231 (182–285)	−15 (−28 to −3)	.0022
Time in high-risk cohort area, min (IQR)	203 (142–255)	52 (34–92)	−134 (−144 to −122)	<.0001
Time in lower-risk areas of ED, min (IQR)	0 (0 to 0)	144 (68–203)	129 (115 to 140)	<.0001
Time to PCR result, min (IQR)	207 (143–301)	322 (249–484)	116 (93 to 140)	<.0001

Note. PCR, polymerase chain reaction assay; MxA, myxovirus resistance protein. CI, confidence interval. ED, emergency department.

a Differences in medians and 95% CI calculated using Hodges-Lehmann estimate.

### Comparison of risk factor-based and FebriDx-based COVID-19 risk triage algorithms, based on subsequent SARS-CoV-2 PCR results

**Fig. 4.**
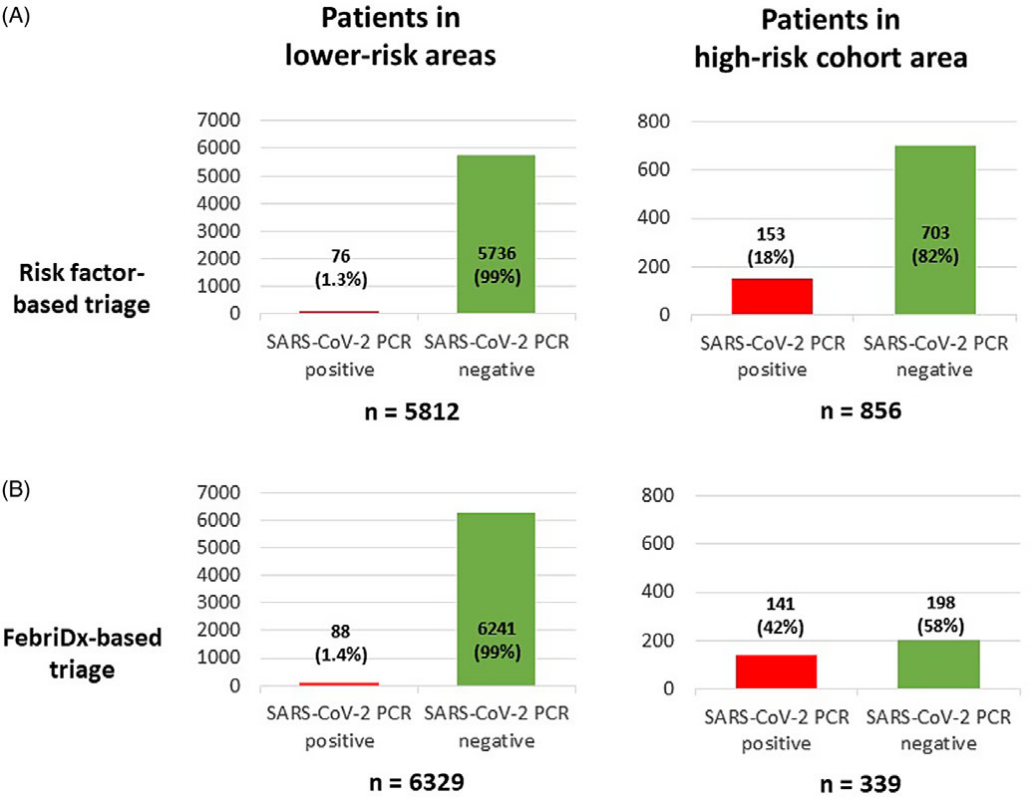
Subsequent SARS-CoV-2 PCR positivity of patients in each area of the emergency department, based on initial risk factor-based triage. (A) Hypothetical situation that would have occurred during the study period if FebriDx testing was not undertaken and patients were therefore not moved from the high-risk cohort area to lower risk areas. (B) FebriDx-based triage, actual situation during study after patients were FebriDx tested and moved from the high-risk cohort area to lower risk areas based on the result. Of the patients in the high-risk cohort area, only those FebriDx tested were included.

Of the 1,321 patients tested using FebriDx (those initially triaged to the high-risk pathway), 856 (65%) were admitted and subsequently had PCR testing for SARS-CoV-2. Of the 14,956 patents who attended ED and did not have risk factors for COVID-19 (those initially triaged to the lower-risk pathway), 5,812 (39%) were subsequently admitted and had PCR testing. Use of the previous risk factor-based triage algorithm (Fig. 1A) would have resulted in all 856 patients remaining in the high-risk cohort area, but only 153 (18%) of these 856 were subsequently PCR positive for SARS-CoV-2. Of the 5,812 patients triaged to the lower-risk areas in ED, 76 (1.3%) were subsequently SARS-CoV-2 positive (Fig. 4A).

Using the study FebriDx-based triage algorithm (Fig. 1B), compared to the risk factor-based algorithm, reduced the numbers of patients managed in the high-risk pathway from 856 to 339 (reduction of 60%; 95% CI, 56%–63%). This allowed effective reconfiguration of clinical areas and other previously suspended ED services to recommence. In addition, the FebriDx-based algorithm increased the proportion of SARS-CoV-2 PCR-positive patients in the high-risk cohort area from 153 (18%) of 856 to 141 (42%) of 339 (difference of 24%, 95% CI, 18%–30%; *P* < .0001). The proportion of SARS-CoV-2–positive patients managed in the lower-risk areas in ED (Fig. 4B) did not significantly change with the FebriDx-based algorithm: 76 (1.3%) of 5,812 versus 88 (1.4%) of 6,329 (difference of 0.1; 95% CI, −0.3 to 0.5; *P* = .638).

### Diagnostic accuracy

The prevalence of SARS-CoV-2 in the high-risk area over the study period was 153 (18%) of 856. Measures of diagnostic accuracy of FebriDx MxA detection of COVID-19, compared to the reference standard of PCR, are shown in Table 3.

**Table 3.** Measures of Diagnostic Accuracy of FebriDx MxA Detection for Identification of COVID-19, Compared to the Reference Standard of PCR Positivity (n = 856)

Variable	No./Total	% (95% CI)
Prevalence of COVID-19	153/856	18 (15–20)
Sensitivity	124/153	81 (74–87)
Specificity	661/703	94 (92–96)
Positive predictive value	124/166	75 (69–80)
Negative predictive value	661/690	96 (94–97)
Positive likelihood ratio	0.81/0.06	13.6 (10.0–18.4)
Negative likelihood ratio	0.19/0.94	0.20 (0.15–0.28)
Overall accuracy	785/856	92 (90–93)

Note. PCR, polymerase chain reaction assay; CI, confidence interval.

Of the 29 patients who tested MxA negative but were PCR positive, 12 (43%) of 28 (missing data in 1 patient) had a low viral load (Ct value of >35 or equivalent); 12 (43%) of 28 (missing data in 1 patient) had a non–COVID-19 primary diagnosis on their discharge summary, and 7 (24%) of 29 had a previous diagnosis of COVID-19 >14 days prior to the admission. Of these 29 patients, 16 (55%) had at least 1 of these factors.

## Discussion

Delays that arise from waiting for confirmatory PCR-based diagnostic testing for COVID-19, coupled with limited side room availability, results in most UK hospitals cohorting patients together based on clinical likelihood of infection.^1^ This results in patients with non–COVID-19–related illness being initially managed within high-risk cohort areas alongside patients with COVID-19. Given that close indoor contact increases transmission rates,^17–20^ this increases the risk of COVID-19 nosocomial acquisition, which hinders the management of the epidemic and carries a high risk of death.^2,3^ At least 14%–24% of patients diagnosed with COVID-19 in UK hospitals have acquired the infection nosocomially,^21,22^ and efforts to reduce transmission are therefore a national priority.

We assessed the clinical impact of the use of the FebriDx PoCT as part of a triage tool for COVID-19 in the ED. We have demonstrated that most patients with risk factors who are triaged to a high-risk cohort area do not, in fact, have COVID-19, even during a period of high prevalence. FebriDx was able to correctly identify the majority of these patients and allowed them to be rapidly moved out of the high-risk cohort area. Without the FebriDx testing algorithm, these patients would have remained in a high-risk cohort area for the duration of their ED stay until admitted or discharged, placing them at risk of nosocomial infection. Moving FebriDx MxA-negative patients out of the high-risk area significantly reduced overall patient numbers there and increased the proportion of PCR-positive patients within that area. This significantly improved departmental flow and allowed the limited number of high-risk patient beds to be used more appropriately.

In our study, FebriDx MxA had a high negative predictive value for COVID-19 (96%; 95% CI, 94%–97%) compared with the reference standard of PCR, which is highly consistent with findings in our previously published diagnostic accuracy study.^11^ This high negative predictive value, despite the high prevalence of SARS-CoV-2 in this study, enables confident decision making in ED, allowing FebriDx MxA-negative patients to be rapidly moved from high-risk to lower-risk areas without waiting for the results of PCR testing. The lower sensitivity of 81% for FebriDx MxA compared with PCR may relate to the study including large numbers of frail elderly patients who did not have pneumonia and had low levels of RNA detected, likely representing persistent viral shedding from past infection.^23^ Although this lower sensitivity resulted in small numbers of patients being moved to a lower-risk area and subsequently testing PCR positive for SARS-CoV-2, these patients likely represent a lower infective risk. Furthermore, the overall proportion of patients with COVID-19 managed in the lower-risk area was unchanged with and without FebriDx testing (1.4% vs 1.3%, respectively).

For effective use in the emergency department, PoCTs must yield rapid results to change patient management procedures.^24^ Hence, even the relatively rapid turnaround times of many PCR-based point-of-care SARS-CoV-2 tests are too long to enable meaningful changes in ED triage and treatment pathways.^25–27^ FebriDx testing gives results within 10 minutes, and we have shown that its rapid use is feasible in an ED setting. A proportion of patients in the high-risk cohort area were not tested by FebriDx for a variety of reasons, including absence of trained personnel or ineligibility, and this reflects the real-world nature of the study. Despite having no additional staff, the ED still tested >60% of patients and moved almost 80% of those with a negative result, demonstrating that the use of a rapid test can lead to a change in patient pathways without additional resources. A similar recent study also used FebriDx as part of a triage algorithm for medical admissions and found its use feasible, with a comparable high negative predictive value that resulted in a significant reduction in the need for isolation rooms.^28^

Our study had several limitations. As a nonrandomized observational study, we cannot be sure that the changes seen following implementation were directly attributable to the FebriDx results, although this seems highly likely. Our study findings should ideally be confirmed in other studies, and health economic evaluation should be performed. Our study was conducted during a period of high prevalence for SARS-CoV-2, and it is uncertain whether the impact and diagnostic accuracy seen in this study would be maintained during periods of low COVID-19 prevalence, when the negative and positive predictive values would increase and decrease, respectively. The need for this test may also be less in this circumstance because there are likely to be fewer patients presenting with high-risk symptoms. In addition, when there is increased cocirculation of other respiratory viruses, the specificity is likely to be reduced. An additional limitation is that most of the patients discharged directly from the ED did not have a PCR test. This could alter the sensitivity and specificity if a different proportion of discharged patients had COVID-19, compared to those admitted. Lastly, although the impact of FebriDx on patient pathways in the ED in this study suggest that its use would be associated with a reduction in nosocomial transmission, we were unable to measure this directly.
