# FebriDx host response point-of-care testing improves patient triage for coronavirus disease 2019 (COVID-19) in the emergency department – ADDENDUM

**DOI:** 10.1017/ice.2022.90

**Published:** 2022-08

**Authors:** Christopher T. Mansbridge, Alex R. Tanner, Kate R. Beard, Florina Borca, Hang T.T. Phan, Nathan J. Brendish, Stephen Poole, Christopher Hill, Michael Kiuber, Robert Crouch, Daniel Waddington, Tristan W. Clark

The following acknowledgment was omitted from the above mentioned article^1^:

We acknowledge and thank all the patients who kindly participated in this study and all the clinical staff at University Hospital Southampton who cared for them. We also acknowledge the Department of Health and Social Care Antimicrobial Research Capital Funding Award (reference NIHR200638).

## References

[1] Mansbridge, C. , Tanner, A. , Beard, K. , Borca, F. , Phan, H. , Brendish, N ., … Clark, T . (2022). FebriDx host response point-of-care testing improves patient triage for coronavirus disease 2019 (COVID-19) in the emergency department. Infection Control & Hospital Epidemiology, 1–8. doi: 10.1017/ice.2021.531 PMC8828393 35094739

